# Development, Suitability and Comprehensibility of a QR Code–Enabled Educational Package to Improve Self‐Care Practices Among Patients With Type 2 Diabetes: A Multimethod Study in Iran

**DOI:** 10.1111/hex.70729

**Published:** 2026-06-18

**Authors:** Solmaz Mohammadi, Mehrnoosh Zakerkish, Ali Montazeri, Zeinab Bahrami, Marzieh Araban

**Affiliations:** ^1^ Reproductive Health Promotion Research Center Ahvaz Jundishapur University of Medical Sciences Ahvaz Iran; ^2^ Diabetes Research Center, Health Research Institute Ahvaz Jundishapur University of Medical Sciences Ahvaz Iran; ^3^ Health Metrics Research Center, Institute for Health Sciences Research ACECR Tehran Iran; ^4^ Faculty of Humanity Sciences University of Science and Culture Tehran Iran; ^5^ Department of Preventive Services, Graduate School of Medicine, School of Public Health Kyoto University Japan; ^6^ Menopause Andropause Research Center Ahvaz Jundishapur University of Medical Sciences Ahvaz Iran; ^7^ Department of Health Education and Promotion, School of Public Health Ahvaz Jundishapur University of Medical Sciences Ahvaz Iran; ^8^ Food and Drug Research Center, Food and Drug Administration Ministry of Health and Medical Education Tehran Iran

**Keywords:** cultural tailoring, health literacy, patient education, QR code, self‐care, type 2 diabetes

## Abstract

**Background:**

Effective type 2 diabetes (T2DM) education must be culturally relevant and accessible to patients with varying literacy levels. In Iran, most materials lack locally tailored, multimedia‐enhanced guidance.

**Objective:**

To develop a culturally tailored, QR code–enabled educational package for T2DM self‐care and perform a preliminary assessment of its suitability, readability, and comprehensibility.

**Methods:**

This multimethod study (February–October 2025) was conducted in two phases. In Phase 1, we developed the package through a focused literature review and semi‐structured interviews with 30 patients and 10 healthcare professionals. Content was synthesized via conventional content analysis. In Phase 2, a multidisciplinary panel of 30 experts assessed the package using the Suitability Assessment of Materials (SAM) and Readability Assessment of Materials (RAM) tools. Additionally, 30 patients evaluated comprehensibility using the Cloze readability test. Inter‐rater agreement was measured using Fleiss' *κ*.

**Results:**

The resulting 20‐page A5 booklet, *Take Control: Your Guide to Effective Diabetes Self‐Care*, is supplemented by 12 QR‐linked digital resources. Content covers diet/lifestyle, foot care, resource utilization, and medication adherence. The overall SAM score was 78.4% (superior suitability), and RAM scores reached the maximum for all components. The mean Cloze score (72.3%) indicated an independent reading level. Expert agreement was strong (Fleiss' *κ* = 0.82).

**Conclusion:**

This QR‐enabled package is culturally appropriate and highly readable for patients with T2DM. While clinical effectiveness was not evaluated, these results support its potential for further testing in routine diabetes care.

**Patient or Public Contribution:**

Patients acted as study participants, providing qualitative data for content development and evaluating the final material's readability. They were not involved in study design, data analysis, or manuscript preparation.

**Abbreviations**
DALYs: DisabIlity‐Adjusted Life Years
HbA1c: glycated hemoglobin
MeSH: Medical Subject Headings
QR: Quick Response
RAM: Readability Assessment of Materials
SAM: Suitability Assessment of Materials
T2DM: Type 2 Diabetes Mellitus
WHO: World Health Organization

## 1 Introduction

Type 2 Diabetes Mellitus (T2DM) is one of the most prevalent chronic metabolic diseases globally, characterized by insulin resistance and/or a relative deficiency of insulin [1]. According to the World Health Organization (WHO), in 2021, over 537 million adults worldwide had diabetes, and this number is projected to increase to approximately 783 million by 2050 [2].

In Iran, the prevalence of T2DM in adults (25 years and older) was reported to be around 10.8% between 1996 and 2023, primarily attributed to population growth and lifestyle changes [3]. In Ahvaz, this prevalence has been reported as high as 15%, exceeding the national average, and is associated with factors such as age, waist circumference, and family history [4]. In 2019, diabetes was identified as the leading risk factor for ischemic heart disease and Stroke, accounting for 14,191 deaths and over 716,000 DALYs (Disability‐Adjusted Life Years) [5, 6]. In 2022, the economic burden of diabetes in Iran reached $2.9 billion, constituting 12% of the country's total health expenditure, higher than the global average (10%), and is mainly driven by cardiovascular and renal complications [7]. A study indicated that less than 1% of women and even fewer men had met global diabetes control goals by 2025 [8].

To prevent T2DM complications, effective disease management heavily relies on sustained self‐care behaviours, including medication adherence, dietary regulation, regular physical activity, foot care, and continuous blood glucose monitoring. However, evidence suggests that Indian patients with T2DM often demonstrate unfavourable self‐care performance [9, 10]. This situation frequently stems from knowledge gaps, lack of motivation, or inadequate social and systemic support [11].

In this context, health literacy acts as a key determinant. Low health literacy is associated with poor understanding of the disease, poor treatment adherence, reduced self‐efficacy, and, consequently, inadequate blood glucose control and lower quality of life [12]. A cross‐sectional study in Iran showed that health literacy and self‐care behaviours collectively explained over 58% of the variance in health‐related quality of life among diabetic patients [13]. Furthermore, socio‐demographic factors such as education level, socioeconomic status, and access to specialized diabetes education also significantly influence patients' self‐care capacity [14].

A substantial body of literature highlights the importance of structured diabetes self‐management education, with programmes varying widely in their development approaches, delivery formats, and theoretical underpinnings [15, 16, 17]. While some initiatives rely on clinician‐led, didactic models, others integrate participatory design, digital platforms, or community‐based peer support [15, 16]. Recent developments increasingly emphasize hybrid, multimodal formats that combine accessible print materials with interactive digital resources to accommodate diverse learning preferences and health literacy levels [17]. However, many existing programmes are developed using standardized, one‐size‐fits‐all frameworks without systematic incorporation of local cultural contexts, dietary patterns, or literacy‐sensitive design principles. This gap underscores the need for educational resources that are not only evidence‐based but also co‐developed with stakeholders to ensure contextual relevance and practical usability.

Despite these findings, existing educational programmes in Iran. primarily focus on therapeutic or isolated aspects of diabetes management and often lack a comprehensive, culturally assimilated, and literacy‐appropriate approach tailored to the target population [18, 19, 20, 21]. Although various educational packages have been developed for other patient groups, such as those with infectious diseases, miscarriages, spinal cord injuries, or the elderly [22, 23, 24, 25], a significant gap remains in the development and validation of theory‐based, culturally congruent, and literacy‐focused educational packages for chronic diseases, particularly T2DM, in Iran. The integration of quick response (QR) codes into printed educational booklets enables linkage to supplementary multimedia materials and may enhance patient engagement while maintaining the practicality of print‐based resources.

In Iran, existing diabetes education has predominantly relied on fragmented, text‐heavy booklets and brief clinical counselling that do not adequately address low health literacy, culturally specific dietary habits, or the need for continuous self‐care reinforcement. Furthermore, overburdened outpatient clinics and systemic resource constraints limit the scalability and follow‐up capacity of these traditional programmes. The QR code–enabled package was specifically designed to bridge these contextual gaps by embedding literacy‐sensitive, culturally tailored multimedia resources directly into widely accessible printed materials. By utilizing the widespread availability of smartphones in Iran, this hybrid model functions effectively in low‐infrastructure settings, offering a scalable, cost‐effective solution that previous isolated educational methods could not deliver.

Therefore, the present study aimed to develop a culturally tailored QR code–enabled educational package for type 2 diabetes self‐care and to conduct a preliminary assessment of its suitability, readability, comprehensibility, and perceived cultural appropriateness among experts and patients. Rather than evaluating behavioural or clinical effectiveness, this study focused on the development and initial assessment of an educational resource that could later be tested in intervention or implementation studies.

## 2 Methods

### 2.1 Study Design

**Figure 1:**
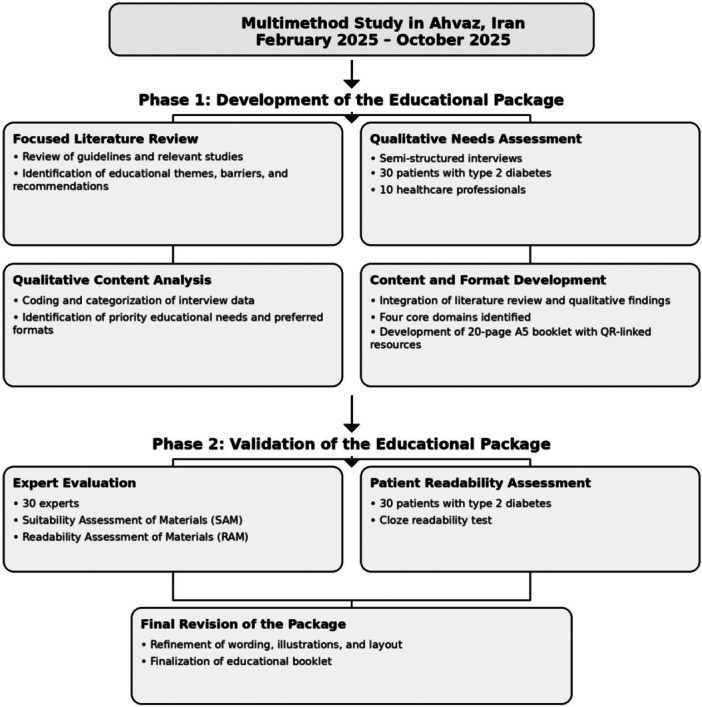
Overview of the two‐phase development and preliminary assessment process of the QR code–enabled educational package for type 2 diabetes self‐care. Phase 1 included a focused literature review, qualitative needs assessment interviews, and development of the booklet content and format. Phase 2 included expert assessment of suitability and readability, patient readability testing, and final revision of the package.

This multimethod study was conducted in Ahvaz, Iran, from February 2025 to October 2025, following ethical approval from the University of Medical Sciences. The study was completed in two phases: (1) development of the educational package through a focused literature review and qualitative semi‐structured interviews with 30 patients with type 2 diabetes and 10 healthcare professionals; and (2) preliminary assessment of the package using expert‐based suitability and readability assessment together with patient‐based readability testing. This was carried out in selected outpatient endocrinology clinics and diabetes care centres in Ahvaz, Iran (Figure 1).

### 2.2 Phase 1: Development of the Educational Package

Phase 1 involved three primary steps:

#### 2.2.1 Focused Literature Review

A focused literature review was conducted to identify key educational themes, effective self‐care strategies, common barriers, and cultural considerations relevant to diabetes education in Iran. The review included current guidelines from the Iranian Ministry of Health, World Health Organization (WHO) documents, and relevant studies on self‐care education for patients with type 2 diabetes mellitus (T2DM). Two researchers independently searched PubMed, Scopus, Web of Science, Cochrane Library, IranMedex, SID, and Google Scholar for literature published between January 1990 and July 2025, with no language restrictions. Search terms were selected in consultation with experts and based on MeSH terms, including ‘type 2 diabetes mellitus’, ‘self‐care’, ‘patient education’, and ‘self‐management’. The PubMed search strategy combined terms related to diabetes, self‐care, patient education, cultural adaptation, and behaviour change. Retrieved records were imported into EndNote X8, and duplicates were removed. Titles and abstracts were screened independently by two researchers. To align with the study's developmental aims, the structured extraction form was explicitly expanded beyond bibliographic details to systematically capture key educational themes, evidence‐based self‐care strategies, common barriers and facilitators, and cultural or linguistic considerations relevant to diabetes management. Extracted data were thematically synthesized and mapped onto core self‐care domains, directly informing the conceptual architecture and content priorities of the educational package. Disagreements were resolved through discussion with a third researcher. Disagreements were resolved through discussion with a third researcher.

#### 2.2.2 Qualitative Needs Assessment

A qualitative needs assessment was conducted using semi‐structured individual interviews with patients with type 2 diabetes and healthcare professionals. The aim was to explore patients' knowledge, beliefs, and practices related to self‐care, the barriers they experienced, and their preferences for receiving educational content. We employed purposive sampling with maximum variation to ensure our patient cohort reflected a broad range of ages, ethnicities, and literacy levels, as well as service setting and professional experience among healthcare providers.

Face‐to‐face interviews were conducted in a private setting at healthcare centres, endocrinology clinics, and diabetes care centres. Interviews followed a semi‐structured guide beginning with open‐ended questions. Example questions for patients included their understanding of diabetes self‐management, challenges in adhering to self‐care practices, helpful types of educational support, and the influence of cultural, social, or economic factors on self‐care. Healthcare professionals were asked about common self‐care challenges among patients, current educational practices, cultural or linguistic barriers, and key elements needed in an effective educational package. Follow‐up probing questions were used when needed. Interviews lasted approximately 45–60 min. Data collection continued until data saturation was achieved, which occurred after interviews with 30 patients and 10 healthcare professionals. Ethical considerations, including informed consent, confidentiality, anonymity, and the right to decline participation or withdraw at any time, were observed. Inclusion and exclusion criteria were as follows:

- Patients: Inclusion criteria were age ≥ 18 years; confirmed diagnosis of Type 2 Diabetes Mellitus for ≥ 6 months; ability to communicate in Farsi; and finally, willingness to participate. Exclusion criteria were presence of cognitive impairment or severe mental illness; significant comorbidities (e.g., advanced renal failure, active cancer) that could interfere with participation; and pregnancy.
- Healthcare Professionals: Inclusion criteria were: (1) employment as endocrinologists, diabetes educators, or nutritionists in selected clinics; (2) ≤ 5 years of clinical experience (to capture recent training perspectives); (3) willingness to participate. Exclusion criterion was unwillingness to provide informed consent.

### 2.3 Qualitative Data Analysis

Qualitative data were analysed using inductive conventional content analysis as described by Elo and Kyngäs [26]. Interviews were transcribed verbatim immediately after each session and analysed concurrently with data collection. The researchers repeatedly read the transcripts to gain an overall understanding of the data and identify meaning units relevant to diabetes self‐care, educational needs, barriers, preferred learning formats, and cultural considerations. These meaning units were condensed, labelled with codes, and grouped according to conceptual and semantic similarities into subcategories and broader categories. The categories were continuously compared and refined until the final thematic structure was developed and used to inform the content and format of the educational package. Coding was performed independently by two researchers, and disagreements were resolved through discussion and consensus with the research team. All semi‐structured interviews were conducted, audio‐recorded, and transcribed verbatim in Farsi (Persian), the participants' native language. Qualitative analysis was performed entirely in Farsi to preserve contextual, linguistic, and cultural nuances. Following theme development, representative participant quotes were independently translated into English by two bilingual researchers, with discrepancies resolved through consensus to ensure accuracy in reporting.

### 2.4 Trustworthiness

Trustworthiness was established based on Lincoln and Guba's criteria of credibility, dependability, confirmability, and transferability [27]. Credibility was enhanced through prolonged engagement with the data, independent coding by two researchers, and regular discussions within the research team throughout the analytic process. Dependability was supported by documenting coding decisions and continuously comparing codes, subcategories, and categories during analysis. Confirmability was strengthened through team review of the analytic process and efforts to minimize researcher bias during coding and interpretation. Transferability was addressed through purposive sampling with maximum variation in patient characteristics and healthcare professionals' backgrounds. Participants were recruited via posters in healthcare centres' waiting rooms and direct invitations from nursing staff during routine visits. In addition, peer debriefing was used to critically examine emerging interpretations and reduce potential bias.

#### 2.4.1 Content and Format Design

The educational content was developed by a multidisciplinary team comprising health education specialists, endocrinologists, a clinical dietitian, a biostatistician, and patient representatives with T2DM. Guided by a participatory, user‐centred design framework, the process followed an iterative cycle of drafting, expert review, and stakeholder feedback. Cultural norms, dietary habits, and religious considerations were systematically integrated at multiple stages. Dietary guidance was contextualized using locally familiar foods, portion‐control strategies aligned with regional eating patterns, and practical meal‐planning tools. Religious considerations, particularly regarding fasting practices and halal dietary principles, were addressed through dedicated sections and clinical‐dietetic consultation. Cultural appropriateness was further ensured by employing plain language, high‐contrast visuals, family‐centred messaging, and locally relevant scenarios. Rather than adhering to a single rigid design theory, a pragmatic, literacy‐sensitive development process was adopted, prioritizing multimodal accessibility and continuous refinement based on Phase 1 qualitative findings and expert consensus.

### 2.5 Phase 2: Suitability, Readability, and Comprehensibility Assessment of the Educational Package

Following the development of the prototype, we conducted a preliminary assessment to evaluate the package's content validity, cultural appropriateness, and technical readability. To ensure an objective evaluation, we recruited a multidisciplinary panel of experts and a separate group of patient validators who were not involved in the initial needs assessment. Inclusion and exclusion criteria for these participants were as follows:

- Expert Panel: Inclusion criteria were: (1) academic or clinical expertise in Health Education, Social Medicine, Endocrinology, Diabetes, Biostatistics, or General Practice; (2) age ≥ 3 years of relevant professional experience; and (3) willingness to complete the evaluation tools. The exclusion criterion was any direct conflict of interest with the study team.
- Patient Validators: Inclusion criteria mirrored those of the Phase 1 patients (age ≥ 18, T2DM diagnosis ≥ 6 months, and Farsi fluency), with the additional requirement of: (4) the ability to read and write in Farsi, which was necessary for the completion of the Cloze readability test. Exclusion criteria remained identical to Phase 1.

We invited a total of 30 experts and 30 patients to participate in this phase. All participants provided written informed consent prior to enrolment, and the protocol received ethical approval from the University of Medical Sciences Ahvaz.

### 2.6 QR Code–Enabled Digital Content: Specification and Organization

We integrated 12 QR codes into the 20‐page booklet to facilitate easier access to multimedia support. Each code links to a specific, culturally tailored multimedia resource hosted on a secure, university‐managed server (no login required). The digital content was designed to complement the corresponding printed page and address diverse literacy levels. The specifications are as follows:

- Format and Duration: Content includes (1) short instructional videos (2–4 min each) demonstrating practical skills (e.g., proper foot inspection, glucometer use, portion control using local dishes); (2) animated infographics (60–90 s) summarizing key concepts (e.g., ‘How insulin works’, ‘Reading food labels in Persian’); (3) audio narrations of the printed text in clear, slow Farsi for low‐literacy users; and (4) downloadable PDF checklists (e.g., weekly meal planner, medication tracker).
- Production Features: All videos feature a native Farsi‐speaking diabetes educator, use simple language (avoiding medical jargon), include Persian subtitles, and incorporate culturally familiar settings (Iranian kitchens, local markets). Visuals use high‐contrast colours and large fonts to accommodate older adults. Audio files are available in both male and female voice options.
- Organization and Navigation: QR codes are labelled with intuitive icons and brief captions (e.g., ‘Scan to watch: How to check your feet’). A master index on the booklet's last page lists all QR‐linked resources by topic and page number. Offline access is supported: once scanned, videos can be downloaded for later viewing without continuous internet.
- Quality Assurance: All digital materials underwent expert review for clinical accuracy, cultural appropriateness, and readability before final integration. The QR links were tested on multiple smartphone models (Android and iOS) to ensure compatibility and fast loading under typical Iranian internet speeds.

This hybrid design ensures that patients with varying digital literacy can benefit: those comfortable with technology can access rich multimedia, while others can rely solely on the printed content without loss of core information. Table 1 provides the specific duration, format, and literacy targets for each digital resource.

**Table 1:** Specifications of QR code–linked digital resources in the educational package.

QR code	Content type	Topic/title	Duration	Format	Target literacy level	Linked page in booklet
1	Instructional Video	How to properly inspect your feet daily	3:15 min	MP4 video with Persian subtitles	Low	Page 8
2	Animated Infographic	Understanding HbA1c and why it matters	1:20 min	Animated GIF + audio narration	Medium	Page 5
3	Audio Narration	Medication adherence: tips for busy days	2:45 min	MP3 audio (male/female voice options)	Low	Page 12
4	Instructional Video	Portion control using Iranian dishes (rice, bread, kebabs)	4:00 min	MP4 video with Persian subtitles	Low	Page 3
5	Downloadable PDF	Weekly meal planner (with local food examples)	N/A	PDF form, printable	All	Page 4
6	Animated Infographic	How insulin works: a simple visual guide	1:45 min	Animated video with voiceover	Medium	Page 6
7	Instructional Video	Proper technique for using a glucometer	3:30 min	MP4 video with close‐up shots	Low	Page 10
8	Audio Narration	Recognizing and managing hypoglycemia	3:00 min	MP3 audio with clear, slow Farsi	Low	Page 11
9	Downloadable PDF	Foot care checklist (daily/weekly tasks)	N/A	PDF checklist, printable	All	Page 9
10	Instructional Video	Reading food labels in Persian supermarkets	2:50 min	MP4 video filmed in local market	Medium	Page 3
11	Animated Infographic	The role of physical activity in blood sugar control	1:30 min	Animated video with simple graphics	Medium	Page 7
12	Downloadable PDF	Medication tracker with reminder prompts	N/A	PDF form, printable	All	Page 13

A preliminary assessment was conducted to evaluate the content validity, cultural appropriateness, comprehensibility, and overall quality of the educational package. A total of 30 experts in health education, social medicine, endocrinology, diabetes care, biostatistics, and general practice were invited to participate in this phase, and written informed consent was obtained in person prior to participation. The experts were then given a PDF version of the educational package together with the relevant evaluation tools to assess its content, readability, and overall suitability. As there is no clear consensus in the literature regarding the optimal number of reviewers required for validation studies [28], a multidisciplinary panel of 30 experts was considered appropriate for this study. At the same time, 30 patients with type 2 diabetes mellitus (T2DM) were included to evaluate the readability and comprehensibility of the material from the perspective of the target population. Written informed consent was obtained from all patient participants before data collection.

The Suitability Assessment of Materials (SAM) tool was to assess content, literacy demand, graphics, layout, and cultural appropriateness via a three‐point Likert scale. A score ≥ 60% was required for approval. This process ensured review and confirmation of the content and face validity based on expert feedback [29].

The Readability Assessment Materials (RAM) Index was used to evaluate the level of content difficulty. This index has three sections: specialized content, writing status (grammar/style), and typographical errors. Each section has specific criteria and assigns scores [30].

### 2.7 Cloze Readability Procedure

Patient comprehensibility was measured using the Cloze Readability Index which was introduced by Wilson Taylor in 1953 at the University of Illinois [31]. The main purpose of this method, as a holistic and Gestalt approach, is to evaluate texts for independent learning, frustration level, and instructional level. Scores were categorized into frustration level (0%–40%), instructional level (41%–60%), or independent level (61%–100%), with the latter indicating the ability to learn without guidance. The opinions of 30 patients were used to evaluate the Cloze Readability Index.

### 2.8 Data Analysis

We analysed quantitative data using SPSS software. Descriptive statistics were used to summarize expert and patient evaluation results. SAM and RAM scores were summarized using frequencies, means, and percentages as appropriate. Inter‐rater agreement for expert assessment of the SAM was evaluated using Fleiss' *κ*. Cloze test scores were calculated as the percentage of correctly completed blanks, and the overall mean score was used to determine the readability level of the material. Qualitative comments provided by experts were reviewed and incorporated into the final revision of the package.

## 3 Results

### 3.1 Findings From Phase 1: Development of the Educational Package

The data analysis from the comprehensive literature review and semi‐structured interviews with 30 patients and 10 healthcare professionals led to the identification of four key areas of self‐care (Table 2): (1) diet and lifestyle management, (2) foot care, (3) utilization of social and healthcare resources, and (4) medication adherence and medical follow‐ups. Participants consistently emphasized the necessity of tailoring educational content to local cultural contexts, literacy levels, and religious dietary practices. As one patient (P4, 58 years) stated: ‘The clinic gives us printed papers full of medical terms. I understand very little and being told to “avoid sweets doesn't help when my family prepares traditional foods like halva and rice daily. I need practical examples I can use at home’. This feedback directly informed our decision to include portion‐control videos using local Iranian dishes rather than generic dietary advice.

**Table 2:** Major categories, subcategories, and illustrative meaning units derived from the qualitative needs assessment interviews with patients with type 2 diabetes and healthcare professionals.

Major category	Subcategories	Illustrative meaning units
Diet and lifestyle management	Culturally appropriate diet; Regular physical activity; Home blood glucose monitoring	Use of traditional foods with modified cooking methods; Difficulty walking because of weather or safety conditions; Need for simple education on reading food labels
Foot care	Daily washing and drying of feet; Regular foot examination; Use of appropriate footwear	Limited awareness of the importance of daily foot examination; Belief that foot problems are unimportant until pain occurs; Difficulty obtaining appropriate shoes because of cost or limited access
Use of social and healthcare resources	Family and community support; Access to healthcare services; Participation in educational classes	Lack of spousal support for dietary adherence; Preference for consulting friends instead of a doctor; Interest in group workshops with other patients
Medication adherence and medical follow‐up	Regular use of diabetes medication; Regular physician follow‐up; Management of medication side effects	Forgetting medication during busy work periods; Concern about becoming dependent on medication; Stopping medication after feeling better; Underestimating the importance of periodic tests

Similarly, a diabetes educator (HCP2) highlighted systemic constraints: ‘We have less than five minutes per patient during follow‐ups. There is no time to properly explain foot inspection or glucometer use. If we could hand out a simple booklet with scannable videos, patients could review it at home, and it would reinforce our brief counseling’.

Common barriers included low health literacy, economic limitations, and inconsistent family support, whereas culturally familiar examples, visual guides, and flexible multimedia formats were identified as key facilitators. Based on these findings, the final educational package was designed as a 20‐page A5 printed booklet integrated with QR‐linked digital resources, titled: ‘Take Control: Your Guide to Effective Diabetes Self‐Care’ (Table 3).

**Table 3:** Evidence‐informed educational recommendations from the focused literature review used to develop the content of the diabetes self‐care educational package.

Self‐care domain	Key recommendations or educational content
Diet and lifestyle management	Limit simple sugars and saturated fats; Encourage consumption of low‐glycemic and high‐fibre foods; Recommend at least 150 min of aerobic physical activity per week
Foot care	Encourage daily foot examination for wounds, redness, or inflammation; Recommend daily washing and thorough drying of the feet; Advise use of cotton socks and appropriate, non‐pointed footwear
Use of social and healthcare resources	Encourage participation in diabetes education classes; Promote family support for dietary adherence and medication reminders; Facilitate connection with support groups and healthcare services
Medication adherence and medical follow‐up	Emphasize regular use of prescribed medications without self‐continuation; Recommend HbA1c testing at least twice per year; Encourage regular physician visits even when symptoms improve

### 3.2 Findings From Phase 2: Preliminary Assessment of the Educational Package

In the preliminary assessment phase, the educational package was evaluated by 30 experts and 30 patients with type 2 diabetes. The expert panel included specialists in health education, social medicine, endocrinology, diabetes care, biostatistics, and general practice. The patient group had a mean age of 51.86 years (SD 13.12), with equal representation of women and men; 28.9% had completed high school education, and 72.2% were married.

Suitability and cultural appropriateness. The overall SAM score was 78.4%, indicating superior suitability of the educational package. All evaluated domains, including content, simplicity, illustrations, layout, appeal, and cultural appropriateness, achieved acceptable ratings. Inter‐rater agreement for expert assessment of the SAM was high (Fleiss' *κ* = 0.82), indicating strong agreement among reviewers. Expert comments resulted in minor revisions to wording and illustrations to further improve clarity and cultural sensitivity.

Readability and editorial quality. According to the RAM, the package achieved the maximum score (6/6) on all three assessed components: specialized content, writing quality, and typographical accuracy, indicating that the material was clear, functional, and free from editorial errors.

Comprehensibility. The mean score on the Cloze readability test was 72.3%, placing the booklet in the independent reading level category. This finding suggests that patients were able to understand the written material without assistance.

## 4 Discussion

This study aimed to develop and preliminarily assess a QR code–enabled educational package to support self‐care among patients with diabetes. The package was developed through a multimethod process that combined a focused literature review with stakeholder engagement, including semi‐structured interviews with patients and healthcare professionals. This participatory approach helped ensure that the package was grounded in the local context and informed by the experiences and needs of its intended users. Similar approaches to health education development have highlighted the value of community and stakeholder involvement in improving the relevance and potential acceptability of interventions [32].

The integration of QR code–enabled digital content with culturally tailored printed materials may offer a flexible and interactive approach to diabetes self‐care education. Such a hybrid format may be particularly useful in populations with diverse literacy levels, varying learning preferences, and unequal access to digital resources [33].

Insights derived from the semi‐structured interviews and literature synthesis highlighted several critical themes that shaped the content and format of the educational intervention. A prominent theme was the importance of understanding the specific needs and perspectives of individuals with diabetes. Tailoring content to the local cultural context and literacy levels was deemed essential for effective communication and engagement, which aligns with previous research emphasizing culturally sensitive education. According to Pambianco et al. (2011), culturally tailored interventions that consider patient beliefs and literacy significantly improve engagement and diabetes outcomes [34]. Similarly, Hill‐Briggs et al. identify health literacy as a fundamental determinant of effective self‐care, underscoring the value of clear and accessible educational materials [35]. Tailored education enhances relevance, which is associated with increased patient motivation and better adherence to self‐care [13]. However, the present study did not evaluate whether the developed package improved motivation, adherence, self‐care behaviours, or clinical outcomes.

Participants expressed a preference for visual, practical, and easy‐to‐follow educational materials, which is in line with evidence supporting the use of multimodal educational strategies in diabetes care [36, 37]. The use of both printed and supplementary digital materials may help accommodate different learning preferences and levels of access. In addition, the involvement of healthcare providers and stakeholders in an iterative feedback process helped improve the accuracy, practicality, and cultural appropriateness of the final package, which is consistent with recommendations for participatory intervention development [38]. At the same time, the barriers identified in this study, including limited resources, language‐related challenges, and variable literacy levels, reflect persistent obstacles to effective diabetes education in real‐world settings [28].

The final educational package, titled Take Control: Your Guide to Effective Diabetes Self‐Care, was developed as a 20‐page A5 booklet designed for visual clarity, logical structure, and ease of use. This format aligns with evidence indicating that well‐organized, multimodal educational materials enhance patient engagement and comprehension [33]. The content is structured around four core domains: diet and lifestyle management, foot care, medication adherence with medical follow‐up, and utilization of social and healthcare resources. These domains reflect established standards for diabetes self‐management education [39]. and are widely recognized as essential components for promoting effective self‐care and reducing diabetes‐related complications [37, 39].

### 4.1 Study Strengths and Limitations

This study has several strengths. The package was developed through a structured multimethod process that incorporated evidence from the literature together with input from patients and healthcare professionals. The use of established assessment tools also strengthened the evaluation of suitability, readability, and comprehensibility. However, some limitations should be acknowledged. The study was conducted in a single setting, and the qualitative and assessment samples were relatively limited, which may affect the transferability of the findings to other populations and contexts. In addition, although the package demonstrated favourable suitability and readability, its effects on self‐care behaviours, glycemic outcomes, and long‐term use were not assessed in the present study. Further research is therefore needed to evaluate its effectiveness in broader and more diverse populations and to explore its integration into routine diabetes care. Additionally, while cultural appropriateness was emphasized throughout development and assessed via the SAM tool, the SAM's cultural subscale comprises only two items (language/logic match and cultural images/examples), contributing a limited proportion to the overall score. Cultural alignment was primarily informed by qualitative needs assessment, iterative expert review, and patient feedback rather than a dedicated, psychometrically validated cultural adaptation instrument. Consequently, the package's cultural appropriateness should be interpreted as preliminary and context‐specific.

## 5 Conclusion

This study developed a culturally tailored QR code–enabled educational package to support self‐care education among patients with type 2 diabetes. The package demonstrated favourable suitability, readability, and comprehensibility, with strong initial indicators of perceived cultural appropriateness based on expert and patient feedback. These findings suggest that the package is appropriate for further testing; however, the present study did not evaluate its effectiveness in changing self‐care behaviours, glycemic control, or long‐term clinical outcomes. Future research should examine patient use of the QR‐linked resources, acceptability in routine care, and effectiveness through pilot intervention or randomized controlled studies.

## Author Contributions

**Solmaz Mohammadi:** conceptualization, investigation, funding acquisition, writing – original draft, methodology, data curation, writing – review and editing. **Mehrnoosh Zakerkish:** methodology, validation, visualization, writing – review and editing, supervision. **Ali Montazeri:** supervision, methodology, validation, writing – review and editing. **Zeinab Bahrami:** writing – review and editing. **Marzieh Araban:** methodology, validation, visualization, writing – review and editing, supervision.

## Ethics Statement

This study was conducted in accordance with the principles of the Declaration of Helsinki. The study protocol was reviewed and approved by the Ethics Committee of Ahvaz Jundishapur University of Medical Sciences, Ahvaz, Iran (Ethics Code: IR.AJUMS.REC.1402.192). Written informed consent was obtained from all participants prior to enrolment in the study.

## Conflicts of Interest

The authors declare no conflicts of interest.

## Data Availability

The data that support the findings of this study are available on request from the corresponding author. The data are not publicly available due to privacy or ethical restrictions.
