# Correction to “Development, Suitability and Comprehensibility of a QR Code‐Enabled Educational Package to Improve Self‐Care Practices Among Patients With Type 2 Diabetes: A Multimethod Study in Iran”

**DOI:** 10.1111/hex.70857

**Published:** 2026-08-28

S. Mohammadi, M. Zakerkish, A. Montazeri, Z. Bahrami, M. Araban, “Development, Suitability and Comprehensibility of a QR Code‐Enabled Educational Package to Improve Self‐Care Practices Among Patients With Type 2 Diabetes: A Multimethod Study in Iran,” *Health Expectations* 29, no. 3 (2026): e70729. https://doi.org/10.1111/hex.70729.

In the “Ethics Statement” section, the Ethics Code was reported incorrectly as “IR.AJUMS.REC.1402.192”. It should be corrected to “IR.AJUMS.REC.1402.191”.

We apologize for this error.

